# Prevalence of SARS-CoV-2 Variants and Disease Outcome of COVID-19 Patients in the Amazonas Region of Peru

**DOI:** 10.4269/ajtmh.22-0739

**Published:** 2023-07-31

**Authors:** Christian J. Campos, Cecilia Pajuelo-Reyes, Luis M. Rojas, Jhony A. De La Cruz-Vargas, Juan R. Tejedo, Rafael Tapia-Limonchi, Pablo Tsukayama, Stella M. Chenet

**Affiliations:** ^1^ Laboratorio Referencial de Salud de Chachapoyas, Dirección Regional de Salud Amazonas, Chachapoyas, Perú;; ^2^ Instituto de Enfermedades Tropicales, Universidad Nacional Toribio Rodríguez de Mendoza de Amazonas, Chachapoyas, Perú;; ^3^ Instituto de Investigaciones en Ciencias Biomédicas, Universidad Ricardo Palma, Lima, Perú;; ^4^ Departamento de Biología Molecular e Ingeniería Bioquímica, Universidad Pablo de Olavide, Seville, Spain;; ^5^ Diabetes and Associated Metabolic Diseases Networking Biomedical Research Centre, Madrid, Spain;; ^6^ Laboratorio de Genómica Microbiana, Facultad de Ciencias y Filosofía, Universidad Peruana Cayetano Heredia, Lima, Perú

## Abstract

Peru was severely affected by COVID-19 with a fatality rate that reached up to 6%. In this study, the relationship between SARS-CoV-2 variants and COVID-19 disease outcome in Amazonas, a region of northeastern Peru, was evaluated. The variants were determined by genomic sequencing, and clinical-epidemiological data were collected from 590 patients between April 2021 and February 2022. There was no association between mortality and hospitalization with any of the variants, but we did find that Omicron is more likely to infect vaccinated and nonvaccinated people. A significant association was also found between unvaccinated patients and hospitalization. Interestingly, in the indigenous population, there were fewer hospitalizations than in the general population. In conclusion, SARS-CoV-2 variants were not associated with the disease outcome in the Amazonas region, and indigenous population were found to be less vulnerable to severe COVID-19 illness.

Since the emergence of SARS-CoV-2, there has been great effort to better understand the evolution of this virus responsible for the COVID-19. Several SARS-CoV-2 variants have been reported and classified by the WHO as variants of concern (VOCs) and variants of interests (VOIs). The VOCs can increase transmissibility and virulence or decrease the effectiveness of available diagnostic tests, vaccines, and therapeutics. On the other hand, VOIs have genetic changes that are predicted or known to affect virus characteristics and cause significant community transmission or multiple COVID-19 clusters.^1^ These variants emerged worldwide and posed a risk for COVID-19 control. In this context, genomic surveillance is necessary to monitor how the virus changes over time and thus understand how these changes affect the characteristics of the virus; this information can be used to assess the impact on public health.^2^

Peru was one of the countries most affected by COVID-19, with more than 3.5 million cases reported by March 2022 and a fatality rate of 5.98%.^3^ In Amazonas, a region in the northeast of the country with a population of 426,806 inhabitants and more than 300 native communities, 43,979 cases were reported with a lethality of 3.03%.^4^ By December 2020, the Lambda variant (C.37) was well described and established in Peru.^5^ A country-wide effort, led by the National Institute of Health in Peru (INS), allowed the sequencing of 704 genomes from Amazonas until February 2022. In this study, we aimed to evaluate the relationship between the prevalence of SARS-CoV-2 variants and disease outcome of COVID-19 patients in Amazonas.

A total of 343 nasopharyngeal swab samples collected in seven provinces of Amazonas between August 28, 2021 and February 4, 2022 by the Regional Directorate of Health (DIRESA) were included in the study (Figure 1A). Specimens were transported in universal transport medium (COPAN Diagnostics Inc., Murrieta, CA), and RNA was extracted on a Lifotronic-PureStar-32 Automated Nucleic Acid Purification System (Lifotronic Technology Co., Shenzhen, China). SARS-CoV-2 detection was performed by real-time reverse transcription polymerase chain reaction with a SARS-CoV-2 Nucleic Acid Detection Kit (Lifotronic Technology Co.) on a QuantStudio 5. Samples reported cycle threshold values < 30 and belonged to patients between the ages 2 and 92 who were hospitalized, had mild symptoms, or were asymptomatic. Variants were determined using the Illumina COVIDSeq RUO kits in a NextSeq500 Sequencing System (Illumina, San Diego, CA). To assemble next-generation sequencing reads and generate a consensus sequence, the Illumina DRAGEN COVID workflow was used. For classifying and naming the variants, the Phylogenetic Assignment of Named Global Outbreak Lineages tool was used. Clinical and epidemiological data were collected from the NOTIWEB registry of DIRESA. Additional sequences (*n* = 247) from Amazonas, reported by the INS from March 2021 to February 2022, were also included. Statistical analysis was performed using Stata version 17.0. The Institutional Review Board of Universidad Peruana Cayetano Heredia approved the project (E051-12-20).

**Figure 1.**
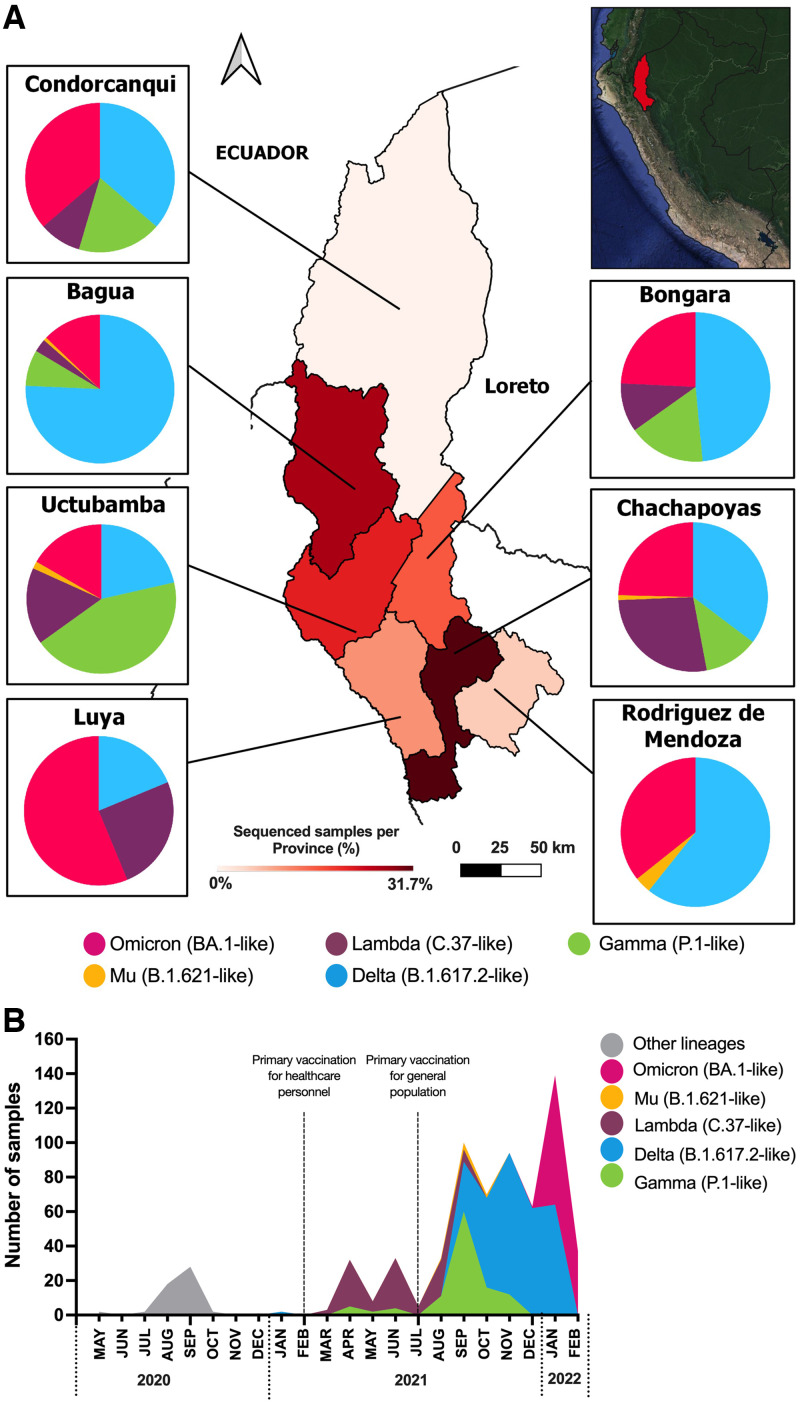
(**A**) Heat map of the samples sequenced in the seven provinces of Amazonas. The pie charts represent the distribution of variants. Variants are represented in pink (Omicron), yellow (Mu), purple (Lambda), blue (Delta) and green (Gamma). The red gradient represents the percentage of sequenced samples per province. (**B**) Prevalence of SARS-CoV-2 variants in Amazonas through time. Vaccination of health workers started in February 2021; in July 2021, vaccination started for the rest of the population, according to age.

According to the results, the median age of the participants (*N* = 590) was 36 (range: 26–50) years, and the most prevalent comorbidities were cardiovascular diseases (4.1%), obesity (2.9%), and diabetes (2.8%) (Table 1). The largest number of sequenced samples were from Chachapoyas (31.7%), and the lowest number were from Condorcanqui (1.9%) (Figure 1A), which correlated with the number of COVID-19 cases during this period.^6^ The first VOI and VOC to emerge were Lambda and Gamma, respectively. Delta was the predominant variant (258 of 590 cases) from August 2021 to January 2022 (Figure 1B). Although an important increase of Mu cases was reported in Colombia and Ecuador,^7^ in Peru, Mu reports were low in 2021.^8^ It is important to note that a fully vaccinated patient who was infected with this variant died without known comorbidity at the time of the study.

**Table 1:** Characteristics of COVID-19 patients sequenced in Amazonas and stratified by SARS-CoV-2 variants

Characteristic	Overall study population*	Delta	Gamma	Lambda	Omicron	
	(*N* = 590)	(*N* = 258)	(*N* = 101)	(*N* = 92)	(*N* = 133)	*P* value†
Age, years (interquartile range)	36 (26–50)	36 (26–50)	36 (27–52)	37 (28.5–53)	35 (27–45)	0.823
Gender (%)						
Male	294 (49.8)	125 (48.5)	51 (50.5)	55 (59.8)	58 (43.6)	0.074
Female	296 (50.2)	133 (51.5)	50 (49.5)	37 (40.2)	75 (56.4)	
Ethnic (%)						
Mestizo	567 (96,1)	245 (95.0)	99 (98.0)	91 (98.9)	126 (94.7)	0.330
Indigenous	23 (3,9)	13 (5.0)	2 (2.0)	1 (1.1)	7 (5.3)	
Occupational group (%)						
Public servers	86 (14.6)	36 (14)	14 (13.9)	12 (13.0)	23 (17.3)	0.893
Others	504 (85.4)	222 (86.0)	87 (86.1)	80 (87.0)	110 (82.7)	
Comorbidities (%)						
Cardiovascular disease	24 (4.1)	12 (4.7)	6 (5.9)	3 (3.3)	2 (1.5)	0.149
Diabetes	14 (2.8)	2 (0.8)	4 (4.0)	5 (5.4)	3 (2.3)	0.058
Obesity	17 (2.9)	5 (1.9)	8 (7.9)	1 (1.1)	3 (2.3)	0.054
Hospitalized (%)						
Hospitalized	39 (6.6)	12 (4.7)	14 (13.9)	10 (10.9)	3 (2.3)	**0.002**
Nonhospitalized	551 (93.4)	246 (95.3)	87 (86.1)	82 (89.1)	130 (97.7)	
Vaccination (%)						
Two doses of vaccine	331 (56.1)	168 (65.1)	38 (37.6)	8 (8.7)	115 (86.5)	**< 0.001**
One dose of vaccine	37 (6.3)	16 (6.2)	12 (11.9)	3 (3.3)	5 (3.7)	
Nonvaccinated	222 (37.6)	74 (28.7)	51 (50.5)	81 (88.0)	13 (9.8)	
Deceased (%)						
Yes	12 (2.0)	3 (1.2)	4 (3.8)	3 (3.3)	1 (0.8)	**0.038**
No	578 (98.0)	250 (98.8)	97 (96.2)	89 (96.7)	132 (99.2)	
Symptoms (%)						
Symptomatic	553 (93.7)	237 (91.9)	95 (94.1)	92 (100)	123 (92.5)	**0.027**
Asymptomatic	37 (6.3)	21 (8.1)	6 (5.9)	0	10 (7.5)	
Reinfection (%)						
Yes	29 (4.9)	10 (3.9)	7 (6.9)	2 (2.2)	9 (6.8)	0.158
No	561 (95.1)	248 (96.1)	94 (93.1)	90 (97.8)	124 (93.2)	

Statistically significant results (*P* < 0.05) are in bold.

* Including 06 cases of the Mu variant.

† Across all SARS-CoV-2 variants (Kruskal–Wallis test, χ^2^).

Omicron was first detected in December 2022 and spread rapidly through the entire population (Figure 1B). The rapid increase of Omicron infections was surprising considering the large number of patients with two doses of the vaccine (115 [86.5%]; *P* < 0.001). However, patients with Gamma had the highest number of hospitalizations (14 [13.9%]; *P* = 0.002), and patients with Mu reported the highest number of fatal outcomes (1 [16.7%]; *P* = 0.038), although there were only six Mu samples included in the study. Delta was the predominant variant, samples included mostly asymptomatic individuals (21 [8.1%]; *P* = 0.027). On the other hand, only 29 (4.9%) patients reported a previous SARS-CoV-2 infection, and the odds of being reinfected with Delta, Gamma, Lambda, Omicron, or Mu were the same. We did not find any association between age, sex, ethnicity, or occupation and likelihood of infection with any of the variants (Table 1).

A multivariate logistic regression was applied to examine the relationship of mortality or hospitalization with SARS-CoV-2 variants. No significant differences were found between variants, but an association of mortality and hospitalization was found with age and diabetes (Table 2). A significant association was also found between hospitalization and vaccination: unvaccinated patients were more likely to be hospitalized than patients with two doses of vaccine (3.01; 95% CI: 1.24–7.30, *P* < 0.014) (Table 2). On the other hand, in the indigenous population, there were fewer hospitalizations (4.4%) than in the general population of Amazonas (6.6%), even though they had the lowest percentage of complete vaccination (52.2%). It should be noted that the median age of the indigenous people was 33 (interquartile range: 21–37) years, which is younger than the general population participants on the study. Furthermore, a previous study suggests that native Amazonian ethnicity might be considered a protective factor given the high incidence of the disease in native communities of Condorcanqui with a relative lower death rate than that reported for the region as a whole,^4^^,^^9^ which emphasizes the need for further studies in these populations. A multivariate logistic regression model was also performed to examine the relationship of vaccination with SARS-CoV-2 variant infections, and it was found that Omicron (177.85, 95% CI: 59.83–528.65, *P* < 0.000) was more likely to infect than Delta (41.40, 95% CI: 15.31–111.94, *P* < 0.000) and Gamma (8.60; 95% CI: 3.02–24.53, *P* < 0.000), even in patients with two doses of the vaccine.

**Table 2:** Logistic regression analysis for COVID-19 patients (*N* = 584)

		Mortality		Hospitalization	
Characteristics		aOR (95% CI)	*P* value	aOR (95% CI)	*P* value
Variants	Omicron (BA.1-like)	Reference category		Reference category	
	Gamma (P.1-like)	1.31 (0.09–19.17)	0.843	1.70 (0.43–6.70)	0.448
	Delta (B.1.617.2-like)	1.49 (0.10–22.21)	0.77	3.82 (0.93–15.57)	0.061
	Lambda (C.37-like)	1.61 (0.11–23.45)	0.724	2.15 (0.48–9.63)	0.313
Vaccination	Two doses of vaccine	Reference category		Reference category	
	One dose of vaccine	5.68 (0.33–96.29)	0.229	1.30 (0.24–7.02)	0.756
	Nonvaccinated	5.18 (0.91–29.41)	0.063	3.01 (1.24–7.30)	**0.014**
Age (years)		1.14 (1.07–1.21)	**< 0.001**	1.05 (1.03–1.07)	**< 0.001**
Comorbidities	Cardiovascular disease	0.25 (0.02–2.43)	0.238	1.10 (0.31–3.92)	0.875
	Diabetes	17.61 (2.11–146.94)	**0.008**	4.11 (1.04–16.19)	**0.043**
	Obesity	N/A	–	4.36 (0.92–20.65)	0.063
Male		1.98 (0.39–10.01)	0.407	1.95 (0.90–4.19)	0.087

N/A = not applicable, not included as covariate in the model. Data are presented as adjusted odds ratios (aOR).

Statistically significant results (*P* < 0.05) are in bold.

Variants of SARS-CoV-2 are the result of natural mutations and the rapid spread of the virus.^10^ For this reason, genomic surveillance is a key tool for tracking transmission or emergence of new variants. Amazonas was one of the regions that performed in situ genomic sequencing, despite the limited genomic sequencing capacity in Peru. Thus, a delay in the introduction of SARS-CoV-2 variants was observed in Amazonas,^8^ perhaps because of the geographic barriers and limited communication pathways. For instance, the Lambda variant was identified at the end of March 2021, almost 3 months after it was first detected in Lima.^5^^,^^11^ This correlated with a peak in the number of cases and deaths in Amazonas from March 2021 to July 2021. It is worth noting that Gamma had also been circulating since the beginning of April 2021, but apparently Lambda spread faster because of its selective advantage of evading the host immune response.^11^ One possibility is that the Lambda held off the expansion of other variants; initially because of a “founder effect” and later due to mutations located in the spike protein that could have been responsible for higher infectivity rates.^11^

Although there was transmission of all SARS-CoV-2 variants in Amazonas, the mortality rate was always below the national average, which could be related to the lower percentage of comorbidities such as hypertension, diabetes mellitus, or obesity in people aged 15 years and older in the region (30.2%) compared with the national average (37.2%) and also to the lower percentage of individuals aged over 65 years in the region (8.7%) compared with the national average (9.3%).^12^ The highest peak of mortality was reached during the highest transmission season between March 2021 to July 2021, when Lambda, Delta, and Gamma were circulating simultaneously. In particular, the introduction of Lambda in several provinces was responsible for the second pandemic wave in the region, in which almost 50 deaths per week were reported.^6^

The arrival of Omicron was associated with the third wave of COVID-19 in Amazonas at the end of 2021, when ∼50% of the population was vaccinated. Although the number of positive cases was twice as high as in the second wave (at the end of 2020), Omicron did not pose a serious health risk as did the other variants of SARS-CoV-2. This observation agrees with previous studies in which Omicron’s higher mutation rate conferred an enhanced transmissibility due to a greater immunological escape compared with other variants. It has been reported that Omicron infection is associated with mild clinical manifestations and lower mortality due to the tropism of this variant for the upper respiratory tract, so it does not represent a serious health risk.^13^ Likewise, positive Omicron patients in Amazonas presented mostly mild to moderate symptoms.

In conclusion, there was no association between a SARS-CoV-2 variant and hospitalization or death in this study; the outcome of COVID-19 disease depended on known risk factors such as age, diabetes, and vaccination. In addition, contrary to what is generally thought regarding vulnerable populations, there were fewer severe COVID-19 cases among the indigenous population than in the rest of the population.
